# Supplemental effect of *Pediococcus acidilactici CNCM I-4622* probiotic on the laying characteristics and calcium and phosphorus metabolism in laying hens

**DOI:** 10.1038/s41598-024-62779-5

**Published:** 2024-05-31

**Authors:** Sureshkumar Shanmugam, Florence Barbé, Jae Hong Park, Eric Chevaux, In Ho Kim

**Affiliations:** 1 Department of Animal Resource and Science, Dankook University, Cheonan-si, Chungnam 31116 South Korea; 2 Lallemand SAS, 19 rue des Briquetiers, 31702 Blagnac Cedex, France

**Keywords:** Probiotic, *Pediococcus acidilactici*, Laying hens, Egg performance, Bone strength, Ca metabolism, Biotechnology, Molecular biology

## Abstract

The close link between intestinal microbiota and bone health (‘gut-bone’ axis) has recently been revealed: the modulation of the amount and nature of bacteria present in the intestinal tract has an impact on bone health and calcium (Ca) metabolism. Probiotics are known to favorably impact the intestinal microbiota. The objective of this study was to investigate the effect of *Pediococcus acidilactici* CNCM I-4622 (PA) on laying performance, egg/eggshell quality, Ca metabolism and bone mineralization and resistance in relatively old layers (50 wks old at the beginning of the experiment) during 14 weeks. 480 Hy Line brown layers were divided into 2 groups (CON and PA: 3 layers/rep, 80 rep/group) and fed with a diet formulated to be suboptimal in calcium (Ca) and phosphorus (P) (− 10% of the requirements). The total egg weight was improved by 1.1% overall with PA, related to an improvement of the weight of marketable eggs (+ 0.9%). PA induced a decreased % of downgraded eggs, mainly broken eggs (− 0.4 pts) and FCR improvement (− 0.8% for all eggs, − 0.9% for marketable eggs). PA also led to higher Haugh units (HU: + 7.4%). PA tended to decrease crypt depth after the 14 weeks of supplementation period in the jejunum (− 25.2%) and ileum (− 17.6%). As a consequence, the VH/CD ratio appeared increased by PA at the end of the trial in the jejunum (+ 63.0%) and ileum (+ 48.0%). Ca and P retention were increased by 4 pts following PA supplementation, translating into increased bone hardness (+ 19%), bone cohesiveness (+ 43%) and bone Ca & P (+ 1 pt) for PA-supplemented layers. Blood Ca and P were respectively improved by 5% and 12% with PA. In addition, blood calcitriol and osteocalcin concentrations were respectively improved by + 83% and + 3% in PA group at the end of the trial, compared to CON group. There was no difference between the 2 groups for ALP (alkaline phosphatase) and PTH (parathyroid hormone). PA significantly decreased the expression of the following genes: occludin in the small intestine, calbindin 1 in the ovarian tissue and actin B in the bone. PA therefore improved zootechnical performance of these relatively old layers, and egg quality. The parallel increase in Ca and P in the blood and in the bone following PA supplementation suggests an improvement of the mineral supply for eggshell formation without impacting bone integrity, and even increasing bone resistance.

## Introduction

Antibiotics are used at sub-therapeutic levels in livestock feed to control the growth of pathogenic bacteria in the gut and to promote growth performance^1^. However, certain antibiotics cause bacterial residues in animals and create major health problems to consumers through the food chain^2^. For this reason, many countries, including South Korea, banned the use of antibiotics as growth promoters (AGPs) in livestock feed^3^. Consequently, due to the increasing pressure on livestock production, various alternative strategies with the potential to replace AGP in animal feed have been proposed and investigated by animal nutritionists. Especially particular attention has been given to the use of prebiotics, probiotics, and organic acids, as alternative AGP, in livestock feed. Various studies suggested that the above-mentioned supplements exert beneficial effects on growth parameters, nutrient digestibility, microbial shedding, and reduce the noxious odor in pigs and poultry.

Probiotics are live microbial feed supplements, which beneficially affect the host animal by improving its microbial balance^4,5^. The suggested modes of action of probiotics *Lactobacillus reuteri, Lactobacillus salivarius,* and *Pediococcus acidilactici* (*P. acidilactici*) are to enhance the growth performance, nutrient absorption, with a higher egg laying rate percentage and an improved feed conversion rate, leading to improved gut health with increased *Lactobacilli* counts in broiler chickens^6–8^, and laying hens^9,10^. In addition, Mikulski et al.^10^ also stated that the probiotic containing *P. acidilactici* strain CNCM I-4622 enhanced the laying hen’s productive performance, increased the egg weight, egg mass output, and eggshell thickness during a 16-week laying period. According to our knowledge, some studies were performed especially using *P. acidilactici* CNCM I-4622 in laying hens^9,10^, but none focused on the phosphorus (P) and calcium (Ca) metabolism in laying hens supplemented with this probiotic. It is then the first study using *P. acidilactici* (PA) CNCM I-4622 in old laying hens. This study aimed at evaluating the effect of this probiotic on the laying performance, nutrient digestibility, egg quality traits, and calcium and phosphorus metabolism in 50 weeks old Hy-Line brown layers during 14 weeks.

## Materials and methods

### Animal care

The animal care and experimental procedures described in this experiment were conducted according to the Animal Welfare Committee guidelines and received the approval of ethics committee of Animal Resource and Science College and Dankook University (DK-1-2040, Cheonan, South Korea). The experiments were performed in accordance with ARRIVE guidelines (https://arriveguidelines.org).

### Source of additive

The probiotic “*Pediococcus acidilactici CNCM I-4622*” (Bactocell^®^; PA) was produced and commercialized by Lallemand SAS (Blagnac, France) and guaranteed to contain a minimum of 1 × 10^10^ cfu viable bacterial cells/g.

### Birds and husbandry management

480 Hy-Line brown laying hens (50 weeks old) of approximately similar body weight (BW) were assigned to two dietary treatment groups in a 14-week trial. The hens were fostered in large double-sided four-tier battery cages with 20 replicates for each treatment (1 bird/cage (45 × 35 × 37 cm)), with 3 adjacent cages representing a replicate. During the experimental period, birds received feed and water ad libitum, with 16 h of daily lighting. The temperature and relative humidity of the air inside the fostering room was maintained at minimum and maximum of 26 °C and 30 °C respectively, and 60% of humidity was maintained until the end of the trial. Hens were allotted into replicates of 3 hens, i.e., 80 replicates/group and 240 birds per group. Feed intake was individually measured, while egg record datasheet reported the laying performance for each replicate of 3 hens. A wire egg collection tray was fixed in front of all cages to avoid eggs from being mixed between replicates.

### Experimental design and animals

Laying hens were randomly allocated into 1 of 2 dietary treatment groups CON—Control (Corn-soybean meal based basal diet) and PA-basal diet supplemented with PA probiotic (2 × 10^12^ CFU *Pediococcus acidilactici* CNCM I-4622/ton of feed during the first 3 weeks of the trial and 1 × 10^12^ CFU PA/ton of feed for weeks 4–14 inclusively). The trial lasted 15 weeks, with one week of adaptation (week 0) and 14 weeks of supplementation. The ingredients and the chemical composition of the experimental diets are shown in Table 1. Basal diets (mash form) were formulated according to NRC^11^ recommendation.

**Table 1:** Composition of laying hen diets (as fed-basis).

Item	Basal diet
Ingredients (%)	
Corn	66.05
Soybean meal	23.28
MDCP	1.00
Limestone (30%fine 70%coarse)	9.2
Salt	0.05
Methionine (99%)	0.12
Vitamin mix^a^	0.10
Mineral mix^b^	0.10
Choline (50%)	0.10
Total	100
Calculated value	
Dry matter, %	89.85
Moisture, %	10.15
Crude protein, %	16.02
Crude fat, %	2.70
Crude fiber, %	3.08
Crude ash, %	4.57
Calcium, %	3.76
Phosphorus, %	0.55
Available phosphorus, %	0.32
Lysine, %	0.75
Methionine, %	0.38
Methionine + cystine, %	0.65
Threonine, %	0.59
Tryptophan, %	0.21
Arginine, %	1.03
Alanine, %	0.84
Aspartic acid, %	1.54
Glutamic acid, %	2.85
Glycine, %	0.64
Proline, %	1.00
Serine, %	0.76
Tyrosine, %	0.59
Isoleucine, %	0.68
Valine, %	0.77
Leucine, %	1.44
Phenylalanine, %	0.79
Histidine, %	0.43
MEn, kcal/kg	2750
Linoleic acid, %	1.55
Vit D, IU	3300

^a^Provided per kg of diet: vitamin A, 8,000 IU; vitamin D3, 3,300 IU; vitamin E, 20 g; vitamin K3, 2.5 g; vitamin B1, 2.5 g; vitamin B2, 5.5 g; vitamin B6, 4 g; vitamin B12, 23 mg; biotin, 75 mg; folic acid, 0.9 g; niacin, 30 g; D-calcium pantothenate, 8 g.

^b^Provided per kg of diet: Fe, 40 g as ferrous sulfate; Cu, 8 g as copper sulfate; Mn, 90 g as manganese oxide; Zn, 80 g as zinc oxide; 1.2 g as potassium iodide; and Se, 0.22 g as sodium selenite.

### Sampling and analysis

#### Laying performance

The percentage of downgraded eggs (dirty, broken, soft, small eggs) (%) and the total and marketable laying rate (%) were recorded per replicate of 3 hens at each day of the trial, then averaged per week. The marketable laying rate was calculated as follows: % total laying rate—% downgraded eggs and the number of marketable eggs as total number of eggs—number of downgraded eggs. The cumulated weight of marketable eggs and downgraded eggs was recorded per replicate of 3 hens at each day of the trial, then averaged per week. The average egg weight was determined by dividing the weight of the collected eggs by the number of eggs laid per replicate. The exported egg mass (all eggs and marketable eggs) was calculated as follows: laying rate (%) × average egg weight. The feed intake was recorded individually weekly and calculated as follows: total of distributed feed each day—refusals at the end of each week, and then averaged per replicate of 3 hens. The feed conversion ratio (FCR) was calculated at every week (1 to 14) for all eggs and for marketable eggs, based on feed intake and egg production data with the following calculation: feed intake/(nb of eggs × average egg weight).

#### Egg quality traits

All the eggs produced on the last day of each week were individually weighed and graded (Ministry of Agriculture, Food and Rural Affairs). The 4 categories recorded for egg size were extra-large (> 68 g), large (61 to 67 g), medium (53 to 60 g), and small (44–52 g) previously described by Mikulski et al.^10^. Then, eggs having medium weight were randomly collected to analyze egg quality parameters. The egg quality traits such as eggshell color, yolk color, eggshell strength, eggshell thickness, the relative weight of egg compartments (yolk, albumen, eggshell), albumen height and Haugh units (HU) were evaluated at the end of weeks 1, 3, 5, 7, 9, 11, and 13 (2-week intervals) on 30 eggs per treatment for each time point. The eggs were randomly collected (30 eggs/treatment) from each group at 5:00 pm, weighed individually using digital precision scale (0.01 g) and brought to laboratory for egg quality analysis. In brief, first, the eggshell color was checked using eggshell color fan (Daeho Co. Ltd). Second, the egg weight, eggshell strength, albumen height, yolk color, Haugh Unit (HU) were measured using fully automatic DET 6000 egg tester (Kyoto, Japan) and noted for statistical analysis. The yolk was then separated from the albumen using a Teflon spoon and placed on the blotting paper towel to remove adhering albumen. Then, the yolk and albumen were stored in clean paper cup. The yolk, albumen and eggshell weights were measured separately using digital Sartorius BCA2201i—1S weigh scale (Gangnam, Korea) and expressed as a percentage of the weight of whole egg (%). To determine eggshell weight, the eggshells were cleaned, and the inner membrane was removed. Then the eggshells were allowed to dry at room temperature. Later, the eggshell thickness was measured at 3 points (rounded end (top), pointed end (bottom), and the middle) using digital micrometer gauge (Baxlo Instrument, Spain) and the mean value was taken as thickness. Above stated internal and external egg quality analysis were done within 24 h of egg collection.

#### Intestinal morphology

10 hens per group and per time point were humanely killed by cervical dislocation at the beginning and end of the experiment for small intestinal morphology analysis in 3 intestinal sections (duodenum, jejunum, ileum), after staining for microscopic observation (LEICA ICC50 E, LEICA, Germany) and imaging. The following parameters were recorded: villus height (VH), crypt depth (CD) and the ratio VH/CD.

#### Nutrient digestibility

Chromic oxide (0.3%) as an indigestible marker was added to the diet of layers at the end of week 13 and provided for about one week until the end of the experiment to measure the nutrient digestibility. Representative feed samples were collected using sterilized plastic bags from each treatment group right after mixing the marker. At the end of week 14, approximately 50 g fresh excreta samples were collected from 20 birds/treatment (1bird/cage) using stainless steel collection tray. Then excreta samples were brought to the laboratory within 30 min and stored at -20℃ to examine the apparent total tract nutrient digestibility (ATTD) of dry matter (DM), nitrogen (N), energy (GE), fat, calcium, and phosphorus. Prior to analysis, all feed and feces samples were placed in a hot air-drying oven for 24 h at 105 °C. Later the samples were grounded to pass 1 mm screen sieve mesh. DM, N, E and fat digestibility was carried out according to the procedure of AOAC^12^. However, ATTD of Ca and P retention digestibility were analyzed as described by Luh Huang and Schulte^13^. The chromium absorption was identified using UV-1201 spectrophotometry. GE was analyzed using Parr 6400 oxygen bomb calorimeter (Parr Instrument Co., Moline, IL, USA) and N was analyzed using TecatorTM Kjeltec8400 analyzer (Hoeganaes, Sweden). The content of calcium and phosphorus in feed sample was determined by the optical emission spectrometry with excitation in the inductively coupled argon plasma in the Optima 2,000 DV camera. The apparent total tract digestibility was calculated using: ATTD (%) = 100 − [(NF/ND) × (CrD/CrF)] × 100], where NF, ND, CrD, and CrF were referred as nutrient concentration in the excreta sample (feces, F), nutrient concentration in the diet (D), chromium concentration in the diet (D) and chromium concentration in the excreta sample (feces, F), respectively.

#### Blood biomarkers

At the beginning and at the end of the experiment, 20 birds were randomly selected from each treatment and blood samples were collected from the brachial vein with sterile K_3_EDTA vacuum tubes (Becton Dickinson Vacutainer Systems, Franklin Lakes, NJ) and stored at 4 °C. For serum analysis, approximately 3 mL blood samples were centrifuged at 4000×*g* for 15 min at 4 °C, and serum calcitriol was assessed using enzyme-linked immunosorbent assay kits (Calcitriol ELISA Kit (OKEH02542), Aviva Systems Biology Corporation, UAS) following the manufacturer’s protocol. Serum concentrations of osteocalcin (OC), parathyroid hormone (PTH), and alkaline phosphatase (ALP) were determined using commercial kits (Immutopics, Inc., San Clemente, CA), the immunoradiometric assay method, and a gamma counter (BioSource International, Camarillo, CA), respectively. The blood minerals (Ca and P) were determined using the commercial kits (MyBioSource, San Diego, CA; BioAssay Systems, Hayward, CA).

#### Bone parameters

At the beginning and at the end of the trial, 20 birds from each treatment were randomly selected to analyze the bone parameters such as hardness, cohesiveness, and bone calcium and phosphorous contents. After thawing the tibias for at least 24 h at 15 °C, they were broken with the Three-Point Bending Test of Animal Bone following the ASABE Standards 2007 [ANSI/ASAE S459 MAR1992 (R2007)] using a Zwick and Roell universal testing machine with a 2.5 kN load cell. The fulcrum was adjusted at 55 mm to get the requested length to bone diameter ratio (greater than 10). The smallest diameter of the tibias was measured. The bones were laid in the test apparatus with the flattest side down, and the force was applied to the midshaft with a crosshead speed of the loading bar of 10 mm/min. The force–deformation curve was read from the texture analyzer. From this curve, the ultimate force required to break a bone was recorded in Newton. To take bone size into account, the value of the ultimate force was divided by the cross-section of the bone (A), which was calculated as the product of π and the square of the radius of the thinnest part of the bone (using the small diameter, see ANSI/ASAE 8.2). This (F/2 × A) was taken as an approximation of the ultimate shear strength. Bones were too thin to measure the outer and inner diameters to get a more exact measurement of ultimate shear strength. Bending strength (force applied when the bone fractures) was automatically derived from the slope of the load/ displacement graph13, and the slope of the load-deformation curve, which is an estimate for the bone stiffness, was derived by the regression between 0.3 and 0.5 mm.

### Gene expression analysis in the small intestine, ovarian tissue, bone and blood

At the end of the experiment, 10 birds/treatment (randomly selected) were slaughtered (by cutting carotid artery) and samples of bones, small intestine and ovarian tissue were isolated from individual hens. The tissue samples were stored in a − 80 °C deep freezer immediately after collection. A portion of each bone, small intestine, ovarian tissue and blood was homogenized in 500 µl of TRIzol reagent (Invitrogen, Carlsbad, CA) using a taco™Prep homogenizer (GeneReach Biotechnology Corp., Taiwan). Total RNA was isolated from the tissue homogenates of each sample according to the manufacturer’s instructions. Immediately after RNA extraction, RNA quality and quantity were determined spectrophotometrically using an ND-1000 spectrophotometer (Nanodrop Technologies, Wilmington, DE) and were assessed using an Automated Electrophoresis, TapeStation system (Agilent, Santa Clara, CA). The purity of the RNA was determined from the ratio of the absorbance at 260 nm to that at 280 nm (A260/A280). 10 hens were sampled per group, but some samples did not have a high concentration of RNA. So, qRT-PCR was performed with only RNA samples having a concentration that could be used for the experiment, the number of samples actually used was then smaller than the number of samples collected: n = 5 for bone gene expression and n = 3 for blood gene expression (vs n = 10 for small intestine and ovarian gene expression). Each sample was analyzed in triplicate. Small intestine samples were collected from duodenum, jejunum or ileum, but as the RNA concentration was not high enough in some samples, the small intestine samples from each intestinal section were combined and qRT-PCR was performed with only RNA samples at the concentration usable for the analysis. The following procedure was applied for mRNA expression analysis:

### cDNA synthesis and RT-qPCR validation

cDNA synthesis on 500 ng of RNAs was performed to validate treatment-dependent gene expression changes using the ReverTra Ace qPCR RT Master Mix (Toyobo; Osaka, Japan). The RT master mix contains reverse transcriptase, RNase inhibitor, oligo dT primer, random primer, MgCl2, and dNTPs. The cDNA synthesis reactions were carried out for 15 min incubation at 37 °C followed by incubation at 50 °C for 5 min, and 98 °C for 5 min using a thermocycler (Bio-Rad Laboratories; Berkeley CA, USA) at the Center for Biomedical Engineering Core Facility (Dankook University, South Korea). As a housekeeping gene, a primer pair of glycerol aldehyde-3-phosphate dehydrogenase (GAPDH) was designed in the Gallus gallus GAPDH sequences of Ensembl (ENSGALG00000014442) and the synthesized cDNA was verified. A positive RT-qPCR reaction was detected by fluorescent signal accumulation, with cycle threshold (CT: the number of cycles required for the fluorescent signal to cross a fixed threshold) being inversely proportional to the amount of the target nucleic acid in a sample, using CFX96 Real-Time PCR System (Bio-Rad Laboratories; Berkeley CA, USA). The GAPDH as an endogenous control (housekeeping gene) was used to normalize the expression level of the gene of interest. Validation of gene expression changes using the comparative 2-ddCT method for mRNA quantification showed CT values for each sample. For each cDNA sample, RT-qPCR validation was performed in triplicates. For statistical analyses, max Ct values were fixed at 35. Delta Ct was calculated by the difference between the Ct of the gene of interest (for a given sample/replicate) and the Ct of GAPDH gene (for a given sample/replicate). Delta Ct was then averaged per sample (n = 3 replicates). For each sample, delta delta Ct were calculated by the difference between delta Ct of the 2 groups, and 2-DDCT (fold change) was then calculated. Gene specific primer sequences are presented in Table 2. Eight genes (mRNA expression analysis) were analyzed in the intestinal tissue: CALB1 (Calbindin 1 Protein Coding gene), ATP2B1 (ATPase Plasma Membrane Ca^2+^ Transporting 1 Protein Coding gene), SLC34A2 (Solute Carrier Family 34 Member 2 Protein Coding gene) and occludin, claudin-1, claudin-5, ZO-1, ZO-2 (genes involved in tight junction proteins). Three genes (mRNA expression analysis) were analyzed in the ovarian tissue: CALB1 (Calbindin 1 Protein Coding gene), ATP2B1 (ATPase Plasma Membrane Ca^2+^ Transporting 1 Protein Coding gene) and SPP1 (Secreted Phosphoprotein 1 Protein Coding gene). Five genes (mRNA expression analysis) were analyzed in the bone tissue: GUSB1 (Glucuronidase Beta Protein coding gene), SLC34A2 (Solute Carrier Family 34 Member 2 Protein Coding gene), CYP24A1 (Cytochrome P450 Family 24 Subfamily A Member 1 Protein Coding gene), VDR (Vitamin D Receptor Protein Coding gene) and ACTB (Actin Beta Protein Coding gene). Two genes (mRNA expression analysis) were analyzed in the blood tissue: CALB1 (Calbindin 1 Protein Coding gene) and FGF23 (Fibroblast Growth Factor 23 Protein Coding gene). CALB1 was therefore analyzed in the small intestine, the blood and the ovarian tissue. ATP2B1 was analyzed in the small intestine and the ovarian tissue. SLC34A2 was analyzed in the bone tissue and the small intestine. CALB, CYP24A1 and VDR are Ca homeostasis-related genes modulated by vitamin D metabolism. ATP2B21 (plasma membrane Ca^2+^-transporting ATPase I) and SPP1 (osteopontin) are genes involved in ossification and in osteocytes/osteoblasts differentiation. SLC34A2 (solute carrier family 34 member 2), GUSB1 (glucuronidase beta 1), ACTB (actin B) and FGF23 (fibrobast growth factor) are genes involved in hormone and growth metabolism. Occludin, claudin-1, claudin-5, ZO-1 and ZO-2 are genes involved in tight junction proteins.

**Table 2:** Gene specific primer sequences.

Genes group	Forward (5ʹ-3)	Reverse (5ʹ–3ʹ)	Amplicon size	Accession number	Target size (bp)
CALB1	CTTACTGAACTGGCCAGGCT	CATTGCCATCTTGATCGTAC	789	NM_205513	121
ATP2B1	CAGGTACTCATGTGATGGAAG	AACTTGCTTAGTTCTGCCCA	3618	NM_001168002	118
SLC34A2	GTAGGAGGTAAAGCAGCAG	ACACCATGCTGACGATGATG	2025	NM_204474	142
SPP1	CCATTTCTGCCAGCTCTGA	TGAGTCTGCTGAAGTGAAGC	795	NM_204535	139
GUSB1	GAGCGTTGCAATGGTGCGA	CCATCGCCTGTAGCAACCA	1974	NM_001039316	108
CYP24A1	ACAACACCATCAATGAGGTC	GACCTCATTGATGGTGTTGT	1527	NM_204979	107
VDR	GAGTTCATCCTGACGGACGA	TCTGCTGCTCCTCCGACAGT	1356	NM_205098	115
ACTB	ACAGATCATGTTTGAGACCT	ACAGATCATGTTTGAGACCT	1128	NM_205518	113

CALB1, calbindin 1; ATP2B1, ATPase plasma membrane Ca^2+^ transporting 1; SLC34A2, solute carrier family 34 member 2; SPP1, secreted phosphoprotein 1; GUSB1, glucuronidase beta, CYP24A1, cytochrome P450 family 24 subfamily A member 1, VDR, vitamin D receptor; ACTB, actin beta.

### Statistical analysis

Laying performance (total laying rate, marketable laying rate, % downgraded eggs/total eggs, total egg weight, weight of marketable eggs, exported egg mass, feed intake and FCR) and egg quality parameters were analyzed by a mixed model with repeated measures where the group, the week and their interaction were set as fixed effects and the laying hen or the replicate, depending on the parameters, as random factor. Nutrient digestibility, parameters of intestinal morphology, bone parameters and blood biomarkers were analyzed by a mixed model applied to the final time with the group as fixed effect and the laying hen or the replicate, depending on the parameters, as random factor. Owing to the low number of replicates for gene expression data, they were analyzed by the non-parametric Mann–Whitney test at each time point (initial/final). Significance level was set at 5% (P ≤ 0.05) and statistical trends were reported at 10% (P ≤ 0.1). Graphics present means and SD per group for each time point. Tables present the estimated marginal means and SEM provided by the mixed model. Statistical analyses were performed using IBM SPSS 26.0 and figures were prepared with Excel.

## Results

### Laying performance

There was no mortality during the trial. The effect of PA supplementation on laying performance is presented in Table 3. There was no overall difference between the 2 groups for the total laying rate and the marketable laying rate, but a significant group × week interaction was noted for the total laying rate (P < 0.01) and the marketable laying rate (P < 0.05). The total laying rate appeared higher in the CON group at weeks 5, 9, 12, 13 and lower in the CON group at week 14, compared to PA. Conversely, the marketable laying rate was higher in PA group at weeks 4, 7, 11, 14 and lower at week 13, compared to CON group. The total egg weight was improved by 1.1% overall with the probiotic, related to an improvement of the weight of marketable eggs (+ 0.9%) (P < 0.001). The exported egg mass of marketable eggs was enhanced with the probiotic (+ 1.0%, P < 0.01). The percentage of downgraded eggs/total eggs, mainly broken eggs, was decreased with the probiotic (− 0.4 pts, P < 0.1). Hens actual individual daily feed intake was constantly around 109 g/hen/day throughout the trial, without any difference between the 2 groups. FCR was improved with the probiotic (− 0.8% for all eggs, − 0.9% for marketable eggs) (P < 0.05).

**Table 3:** The effects of dietary probiotics supplementation on the laying performance for the 2 groups (CON, PA) per week and for the trial period (wk 1–14).

Mean	Group/week	0	1	2	3	4	5	6	7	8	9	10	11	12	13	14	1–14	SEM	Group	Week	Group × week
% total laying rate	CON	98.75%	98.10%	99.52%	99.70%	97.38%	99.94%	98.81%	98.75%	99.70%	100.30%	97.62%	98.81%	98.33%	100.12%	98.04%	98.94%	0.14%	0.151	< 0.001	0.003
	PA	99.05%	98.15%	98.69%	99.23%	98.15%	98.81%	98.69%	99.58%	98.75%	98.99%	97.20%	99.40%	97.20%	98.69%	99.52%	98.65%				
% marketable laying rate	CON	97.36%	97.37%	98.09%	98.15%	95.98%	98.75%	97.66%	97.30%	98.51%	98.74%	96.34%	97.00%	96.56%	98.76%	96.71%	97.60%	0.23%	0.800	< 0.001	0.015
	PA	97.85%	97.25%	97.90%	97.97%	97.25%	97.78%	97.96%	98.63%	97.95%	98.28%	96.46%	98.51%	96.11%	97.55%	98.46%	97.68%				
% downgraded eggs/total eggs	CON	1.39%	0.73%	1.43%	1.56%	1.40%	1.19%	1.15%	1.45%	1.19%	1.56%	1.28%	1.81%	1.77%	1.36%	1.33%	1.34%	0.14%	0.072	0.278	0.664
	PA	1.19%	0.91%	0.79%	1.26%	0.91%	1.03%	0.73%	0.96%	0.80%	0.71%	0.74%	0.90%	1.09%	1.14%	1.07%	0.97%				
Total egg weight (g)	CON	61.15	60.66	60.79	61.60	61.74	61.13	61.36	61.98	61.32	61.00	61.90	61.68	62.14	62.24	63.06	61.62	0.08	< 0.001	< 0.001	0.162
	PA	61.28	60.80	61.74	62.58	62.63	61.32	61.80	62.31	62.39	61.52	63.00	62.38	63.03	63.19	63.48	62.30				
Weight of marketable eggs (g)	CON	61.66	60.86	61.21	62.20	62.18	61.53	61.80	62.50	61.70	61.54	62.31	62.28	62.67	62.71	63.53	62.07	0.05	< 0.001	< 0.001	0.160
	PA	61.70	61.12	61.99	63.06	62.90	61.71	62.09	62.70	62.64	61.74	63.23	62.67	63.44	63.60	63.84	62.62				
Weight of downgraded eggs (g)	CON	28.00	30.77	29.37	25.88	28.84	32.10	28.77	29.83	28.61	24.66	28.68	28.74	29.49	29.57	28.69	28.69	0.75	0.758	0.879	0.495
	PA	29.45	24.80	28.82	28.70	29.35	29.63	28.68	32.07	32.21	29.27	30.15	30.11	23.70	27.81	30.09	29.05				
Exported egg mass—all eggs (g)	CON	68.15	63.87	69.34	68.44	68.32	69.56	66.75	69.06	67.93	66.92	65.70	69.32	69.54	69.09	69.01	68.09	0.66	0.142	< 0.001	0.559
	PA	68.46	64.60	65.05	69.69	66.46	66.44	65.18	68.42	66.57	65.17	65.13	67.56	66.03	68.27	69.96	66.72				
Exported egg mass—marketable eggs (g)	CON	60.02	59.27	60.07	61.04	59.70	60.77	60.33	60.79	60.78	60.79	60.04	60.43	60.52	61.94	61.44	60.58	0.16	0.009	< 0.001	0.185
	PA	60.37	59.47	60.70	61.78	61.18	60.32	60.81	61.82	61.37	60.68	61.01	61.75	60.99	62.03	62.85	61.19				
FCR—all eggs	CON	1.801	1.832	1.800	1.773	1.813	1.783	1.798	1.779	1.785	1.785	1.809	1.794	1.789	1.754	1.767	1.790	0.004	0.016	< 0.001	0.140
	PA	1.795	1.829	1.790	1.757	1.773	1.799	1.788	1.759	1.772	1.792	1.784	1.761	1.785	1.751	1.729	1.776				
FCR—marketable eggs	CON	1.812	1.840	1.814	1.784	1.827	1.793	1.807	1.791	1.796	1.799	1.822	1.811	1.807	1.766	1.778	1.802	0.005	0.013	< 0.001	0.202
	PA	1.805	1.837	1.797	1.767	1.782	1.807	1.793	1.766	1.780	1.799	1.792	1.770	1.793	1.760	1.738	1.785				

### Egg quality traits

Egg quality traits are presented in Table 4. PA group presented a trend for higher eggshell color value (i.e., darker eggshell) than CON group (P < 0.1), while there was no difference between the 2 groups for yolk color. Eggshell strength appeared also similar between the 2 groups, while there was a group × week interaction for eggshell thickness (P < 0.05): PA group presented thicker eggshells at weeks 5 and 7 than CON group. Egg freshness (Haugh units) was improved in the probiotic group (+ 7.4% overall of Haugh units value compared to control group: P < 0.001), particularly at weeks 7, 9, 11 and 13 (group × week interaction: P < 0.1). There was a trend for higher relative yolk weight in the probiotic group (+ 0.6 pts overall compared to control group: P = 0.1). The relative albumen weight was higher at week 5 and lower at week 9 in PA group compared to control group (group × week interaction: P < 0.001), while the relative eggshell weight was not different between the 2 groups.

**Table 4:** The effects of dietary probiotics supplementation on egg quality traits for the 2 groups (CON, PA) every 2 weeks and for the trial period (wk 1–13).

Mean	Group/week	1	3	5	7	9	11	13	1–13	SEM	Group	Week	Group × week
Eggshell color	CON	13.37	13.50	13.57	12.87	12.60	12.93	11.20	12.78	0.12	0.099	< 0.001	0.550
	PA	13.40	13.73	13.90	12.60	12.80	13.30	12.10	13.07				
Yolk color	CON	9.20	9.17	9.07	8.57	8.90	8.67	8.83	8.86	0.06	0.841	0.005	0.241
	PA	9.03	8.83	9.03	8.97	8.83	8.60	8.93	8.87				
Eggshell strength (kg/cm^2^)	CON	4.30	4.46	4.55	4.01	4.06	4.00	4.32	4.24	0.08	0.488	0.015	0.523
	PA	4.47	4.65	4.15	3.96	4.31	3.82	4.05	4.16				
Eggshell thickness (× 10^–2^ mm)	CON	40.10	38.69	38.51	35.06	40.18	39.92	39.08	38.57	0.25	0.344	< 0.001	0.048
	PA	39.07	38.18	39.74	37.36	39.39	38.72	40.04	38.91				
Haugh units	CON	92.7	89.5	91.4	81.8	88.1	81.3	81.3	85.5	0.89	< 0.001	< 0.001	0.069
	PA	93.9	91.0	93.5	89.0	102.6	87.1	87.4	91.8				
Yolk weight (%)	CON	27.1%	26.0%	26.0%	26.9%	25.9%	26.8%	25.6%	26.2%	0.3%	0.102	0.187	0.137
	PA	27.8%	28.7%	26.7%	26.7%	25.0%	26.5%	27.3%	26.8%				
Albumen weight (%)	CON	54.6%	55.0%	54.7%	55.4%	57.0%	56.5%	55.4%	55.6%	0.4%	0.257	0.384	< 0.001
	PA	53.2%	57.0%	56.3%	55.7%	51.8%	54.7%	54.0%	55.0%				
Eggshell weight (%)	CON	15.2%	14.6%	13.8%	14.5%	15.0%	14.6%	14.9%	14.6%	0.2%	0.232	< 0.001	0.348
	PA	15.6%	14.3%	13.7%	14.7%	14.9%	15.8%	15.7%	14.8%				

### Intestinal morphology

**Figure 1:**
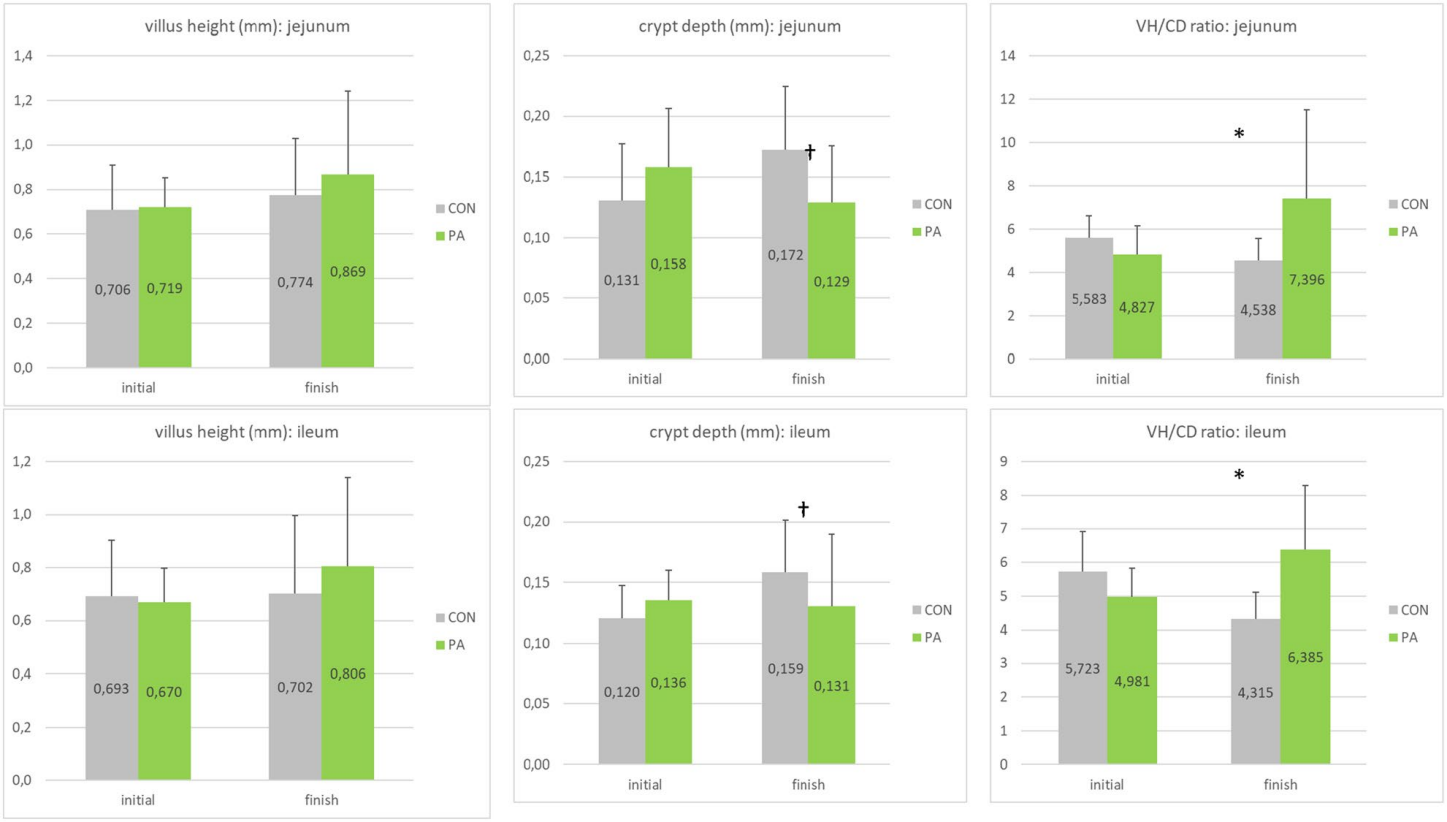
Parameters of intestinal morphology (villus height—VH, crypt depth—CD, VH/CD ratio) for each intestinal section (jejunum: top and ileum: bottom) and for the 2 groups (CON, PA) at start and end of the trial (mean ± SD, *P < 0.05; ^†^P ≤ 0.1).

The effect of PA supplementation on parameters of intestinal morphology of laying hens is shown in Fig. 1. There was no group effect in villus height, crypt depth and VH/CD ratio in the duodenum. There was no effect of the probiotic on villus height in the jejunum and ileum, whereas the probiotic tended to decrease crypt depth after the 14 weeks of supplementation period in the jejunum (− 25.2%, P < 0.1) and ileum (− 17.6%, P < 0.1). As a consequence, the VH/CD ratio appeared increased by the probiotic at the end of the trial in the jejunum (+ 63.0%, P < 0.05) and ileum (+ 48.0%, P < 0.05).

### Apparent total tract nutrient digestibility (ATTD)

**Figure 2:**
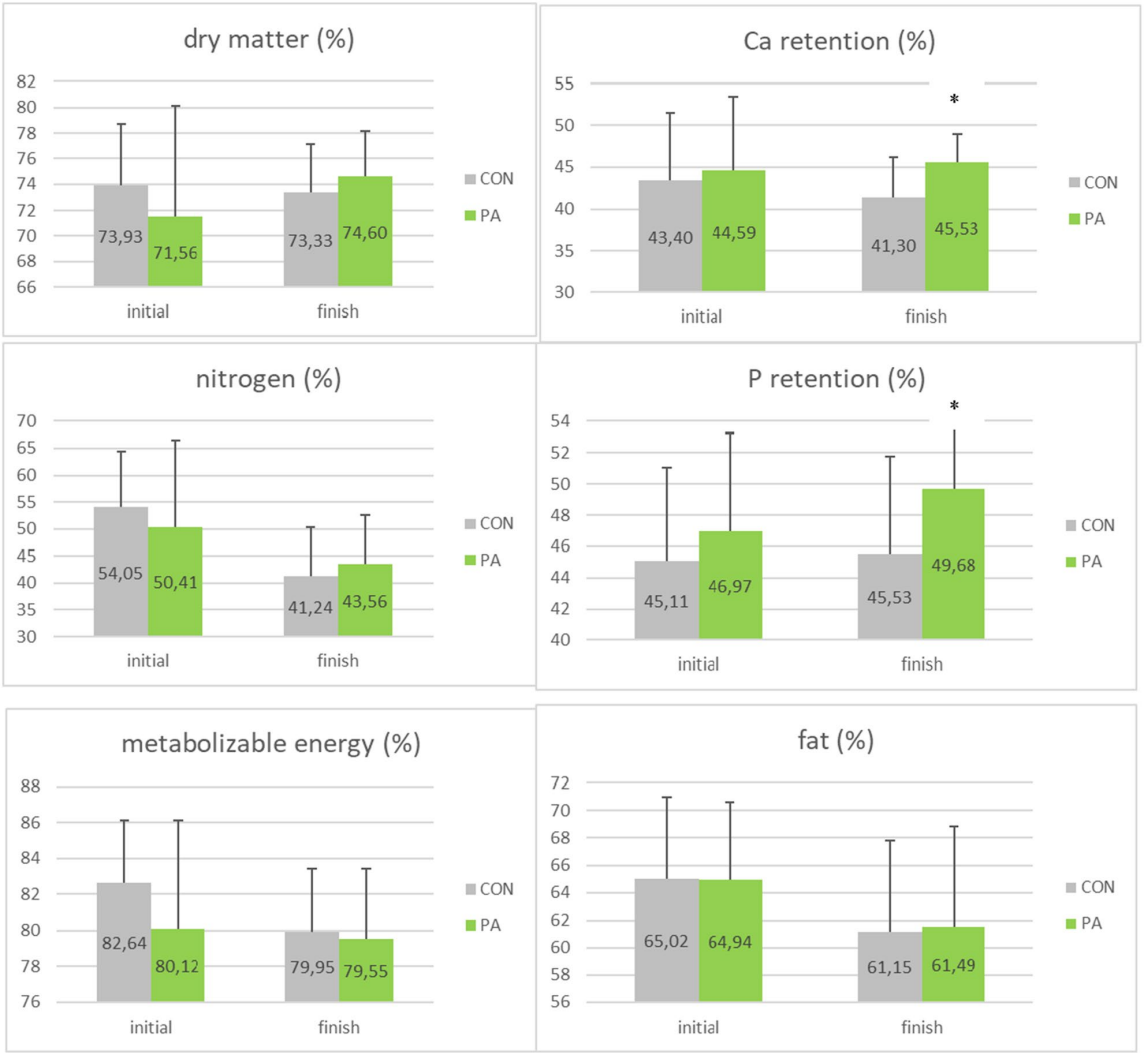
Apparent total tract nutrient digestibility (ATTD) for the 2 groups (CON, PA) at start and end of the trial (mean ± SD, *P < 0.05).

The effect of PA supplementation on nutrient digestibility is shown in Fig. 2. There was no group effect for the ATTD of dry matter, nitrogen, metabolizable energy and fat, while Ca and P retention was significantly increased by 4.2 pts and 4.1 pts, respectively (P < 0.05) at the end of the trial in PA group compared to CON group.

### Blood biomarkers

**Figure 3:**
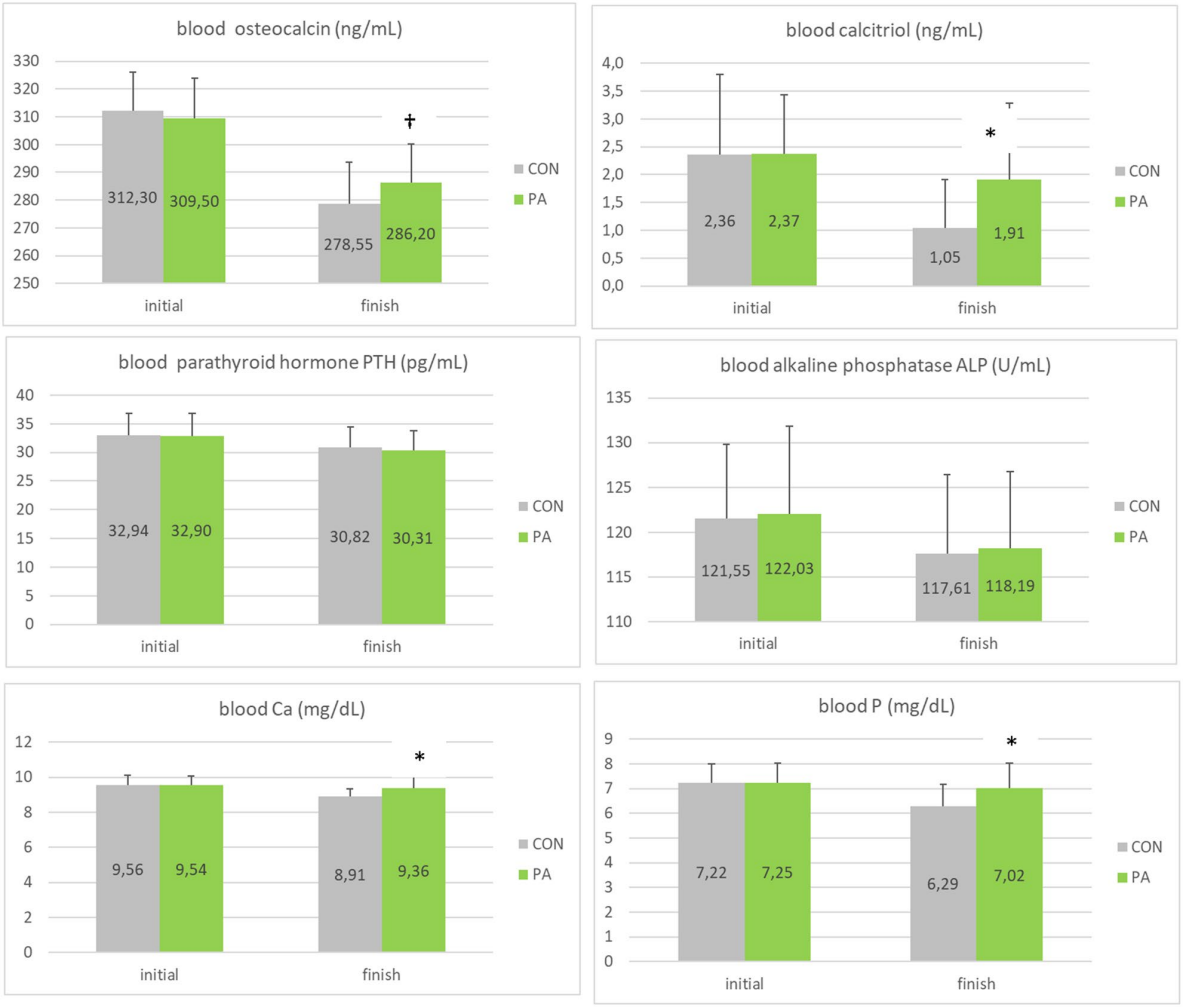
Blood biomarkers for the 2 groups (CON, PA) at the start and end of the trial (mean ± SD, *P < 0.05; ^†^P ≤ 0.1).

The effect of PA supplementation on blood biomarkers related to Ca metabolism (osteocalcin, calcitriol, parathyroid hormone (PTH), alkaline phosphatase (ALP), Ca and P) at the start and end of trial is shown in Fig. 3. Blood Ca and P concentrations were respectively increased by + 5% (P < 0.05) and + 12% (P < 0.05) in PA group at the end of the trial, compared to CON group. Blood calcitriol and osteocalcin concentrations were respectively improved by + 83% (P < 0.05) and + 3% (P = 0.1) in PA group at the end of the trial, compared to CON group. There was no difference between the 2 groups for blood PTH concentrations and for blood ALP activity.

### Bone parameters

**Figure 4:**
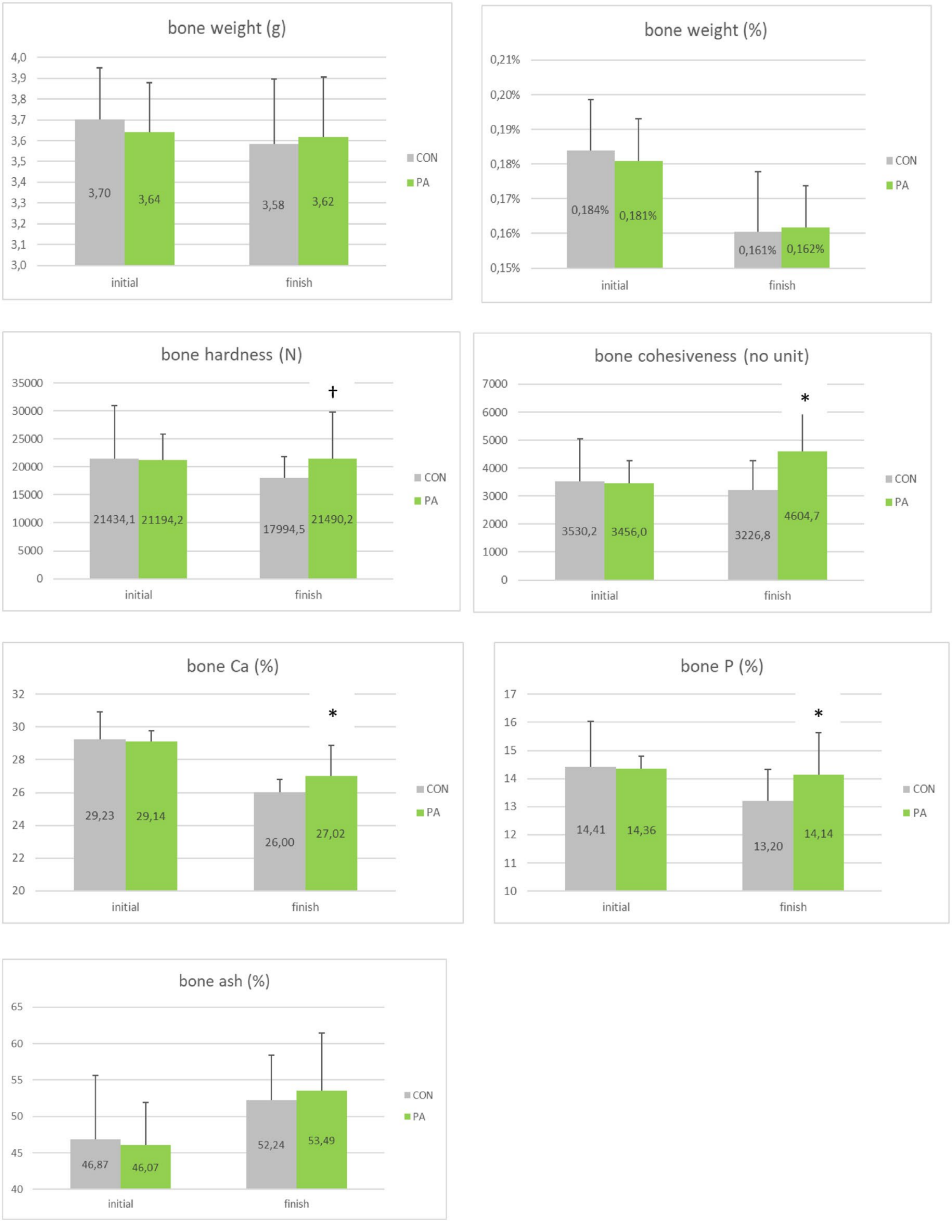
Bone parameters for the 2 groups (CON, PA) at the start and end of the trial (mean ± SD, *P < 0.05; ^†^P ≤ 0).

The effect of PA supplementation on bone parameters (weight, hardness, cohesiveness, Ca, P, ash) at the start and end of trial is shown in Fig. 4. The bone resistance was improved in the PA group at the end of the trial, compared to the control group (bone hardness: + 19%, P ≤ 0.1; bone cohesiveness: + 43%, P < 0.05), while bone Ca, P and ash were respectively increased by + 1pt (P < 0.05), + 0.9pt (P < 0.05) and + 1.2pt (NS) in the PA group at the end of the trial, compared to the control group. However, bone weight was not different between the two treatment groups.

### Gene expression in the small intestine, ovarian tissue, bone and blood

The effects of PA supplementation and time (start and end of the trial) on gene expression (fold change) in the different hens’ organs (small intestine, ovarian tissue, bone and blood) are shown in Table 5. At the end of the trial, PA significantly decreased the expression of occludin-coding gene in the small intestine (P < 0.001) and tended to decrease calbindin 1-coding gene in the ovarian tissue (P < 0.1) and actin B-coding gene in the bone (P < 0.1). There was no difference between the 2 groups for the expression of the other genes studied in these organs and for the genes expressed in blood.

**Table 5:** The effects of dietary probiotics supplementation on gene expression (fold change) in the different organs for the 2 groups (CON, PA) at the start and end of the trial.

Organ	Gene	Initial		Final	
		CON	PA	CON	PA
Small intestine	CALB1	1.341	1.322	1.706	0.353
	P-value	1.000		0.143	
	ATP2B1	1.117	1.636	0.500	0.568
	P-value	0.009		0.631	
	SLC34A2	1.476	1.371	0.117	0.129
	P-value	0.912		0.190	
	Occludin	1.251	1.588	3.015	0.166
	P-value	1.000		< 0.001	
	Claudin-1	1.577	1.577	1.870	2.048
	P-value	0.529		0.684	
	Claudin-5	1.747	1.123	2.408	1.308
	P-value	0.912		0.165	
	ZO-1	1.500	1.862	1.213	1.209
	P-value	0.353		0.912	
	ZO-2	1.383	1.614	1.374	1.101
	P-value	0.529		0.912	
Ovarian tissue	CALB1	5.684	7.414	912.877	191.029
	P-value	0.912		0.075	
	ATP2B1	1.087	0.996	2.535	1.211
	P-value	0.529		0.123	
	SPP1	3.012	2.614	16.100	380.621
	P-value	0.912		0.190	
Bone	GUSB1	1.779	1.389	15.559	2.760
	P-value	0.841		0.421	
	SLC34A2	3.430	0.700	18.728	25.860
	P-value	0.421		0.690	
	CYP24A1	3.320	0.798	153.501	1.799
	P-value	0.548		0.310	
	VDR	1.487	2.307	5.567	6.990
	P-value	0.548		1.000	
	ACTB	1.256	0.932	4.584	1.065
	P-value	0.690		0.056	
Blood	CALB1	1.279	1.279	4.144	0.714
	P-value	1.000		0.400	
	FGF23	1.241	1.241	1.315	5.842
	P-value	1.000		1.000	

## Discussion

In recent years, a lot of research studies have presented compelling evidence explaining the role of probiotics in improving poultry output, particularly broiler chicks and laying hens. The close link between intestinal microbiota and bone health (‘gut-bone’ axis) has recently been revealed in humans, poultry and other animals: the modulation of the amount and nature of bacteria present in the intestinal tract has an impact on bone health and calcium (Ca) metabolism^14–18^. The objective of this study was therefore to investigate the effect of the probiotic strain PA on laying performance, egg/eggshell quality, Ca metabolism and bone mineralization and resistance in relatively old layers (50 wks old at the beginning of the experiment). Gene expression and blood concentration of relevant biomarkers related to Ca metabolism have been measured in the current study to improve the understanding of the mechanisms involved. Previously, Mikulski et al*.*^10^ found that using *P. acidilactici* probiotic increased laying rate and feed efficiency by 2.8%. In the present trial, there was no overall difference between the 2 groups (control/*P. acidilactici*) for the total laying rate and the marketable laying rate, but egg weight, FCR and egg quality (egg freshness (HU), % of downgraded eggs) were favorably affected by the supplementation of PA in these relatively old layers. The positive effect of PA reported in the current study on laying performance and egg quality was previously reported by Awaad et al.^19^, Demey et al.^20^, Denev et al.^20^, Mikulski et al.^10,21^ and Quarantelli et al.^9^. It must be noted that the laying performance observed in this trial was already optimal: > 97% of laying rate. However, these performance levels are in accordance with the historical background of the experimental farm where the trial was conducted. These overall good laying performances could explain the absence of expected differences on eggshell strength, eggshell thickness and relative eggshell weight. In fact, it has been described that the percentage of eggshell, the eggshell strength, thickness and density decrease (and the eggshell deformation increases) in relation to the age of hens and repeated laying cycles^22^. It could be hypothesized that a potential difference in terms of eggshell quality would have been depicted if these hens would have been more challenged in terms of laying performance.

Nowadays, the industry aims to extend egg laying until hens are 100 weeks old or longer (from 65 to 70 weeks old, currently) to make egg production more sustainable. However, intensive egg production challenges hen health and particularly bone metabolism as eggshell formation mobilizes large amounts of calcium from the skeleton, inducing a severe form of osteoporosis and bone fractures. Moreover, the high laying performance of today’s laying hens places enormous demands on their mineral metabolism, inducing bone resorption and weakening^23–26^. Laying hens supply calcium to their eggshells via a process of dynamic bone formation and resorption called bone remodeling. As hens age, their bone becomes weak and brittle due to an imbalance in remodeling, resulting in bone disorders^27^. While Dunn et al*.*^28^ suggest that the selection for increased persistency of egg production may not adversely affect bone quality, the negative effects of hens’ age and extended laying cycles on laying performance, egg/eggshell quality (albumen height and HU, eggshell proportion, thickness and resistance, vitelline membrane strength, cuticle cover), blood parameters associated to bone metabolism, and bone health and mineralization (ash, bone weight and resistance, keel bone damages and fractures) have been extensively studied^29–34^. Bar et al.^35^ reported significantly lower eggshell weight and density in old than in young hens, while de Mezenes et al.^36^ underlined a decrease of albumen height, Haugh units and eggshell percentage with the age of laying hens (35–50 weeks). The marked decrease in eggshell breaking strength (25% reduction) with age is not only explained by the reduction in eggshell thickness (6–10% reduction) but also by abrupt changes in eggshell mineral content and eggshell ultra- and microstructure characteristics, occurring in older hens, along with a decrease in the amount of cuticle and internal egg quality parameters (egg albumen height)^33,37^. Zhang et al.^33^ specified that disturbed regulation of calcium metabolism and uterine expression of ion transporters, especially for HCO3- exchange, in aged laying ducks likely contribute to age-induced-ultrastructural deterioration of the eggshell. The age-related decline in eggshell thickness and eggshell breaking strength is also related to an elevation of egg weight and egg size over time, while hen exports a similar Ca for eggshell mineralization^23,38^. Therefore, the risk of rejected (broken) eggs and osteoporosis increases with advancing hen age, stressing the need to support a normal Ca metabolism. Besides egg and eggshell quality, the age-induced osteoporosis, associated to elevated egg production, leads to increased keel bone damage and fractures and has significant negative impact on animal welfare and production performance. Bone fractures at the end of the laying cycle is indeed one of the most pressing welfare problems in the egg industry^27,39,40^. Most of the Ca source comes from the diet (60–75%) but the medullary bone typically supplies Ca (25–40%) when dietary sources are exhausted, notably during the night^41^. The bone is then reformed when the circulating Ca is sufficient. Miller et al.^42^ established a relationship between hen production status and serum Ca and P levels, which was mediated by shell calcification activity, bone resorption and remineralization, intestinal absorption of minerals and muscular activity. Vitamin D is the major factor regulating intestinal transport and 1.25(OH)2D3 (calcitriol, which is the active form of vitamin D3) was demonstrated to regulate and stimulate both intestinal transport routes of Ca: paracellular and transcellular routes^43,44^. Moreover, 1.25(OH)2D3 has been shown to decrease in aged laying hens^45^. The regulation of Ca and P homeostasis involves different organs (parathyroid glands, kidneys, small intestine, bone and blood). In the current study, Ca and P retention was increased by 4 pts following PA supplementation, translating into increased bone hardness (+ 19%), bone cohesiveness (+ 43%) and bone Ca & P (+ 1 pt, P < 0.05) for PA-supplemented layers. Blood Ca and P were respectively improved by 5% and 12% with PA. Kwiatkowska et al*.*^46^ compiled the results of studies concerning the positive effect of different additives (vitamin D, probiotics, prebiotics, symbiotics) on the accumulation of calcium level in the bones of broilers.

Regarding intestinal morphology, PA tended to decrease crypt depth after 14 weeks of supplementation in the jejunum (-25.2%, P < 0.1) and ileum (-17.6%, P < 0.1). As a consequence, the VH/CD ratio appeared increased by the probiotic at the end of the trial in the respective compartments: jejunum (+ 63.0%, P < 0.05) and ileum (+ 48.0%, P < 0.05). These results confirm the positive effect of PA on intestinal morphology, previously described by Temim et al.^47^. One of the reported effects of probiotics in laying hens is to modify the intestinal morphology, having potential positive impact on nutrient and minerals absorption and digestibility^48^. In the current study, there was no group effect for the apparent total tract digestibility of dry matter, nitrogen, energy and fat, while Ca and P retention were significantly increased by 4.2 pts and 4.1 pts, respectively at the end of the trial in PA group compared to the control group. These values are in the same range of values reported by Mikulski et al.^10^ for Ca and P retention, which were increased respectively by 4.8 pts and 1.5 pts with the same probiotic strain. However, at the end of the trial, layers fed probiotic supplement have significantly increased (P < 0.05) calcium and phosphorus digestibility. Similarly, Park et al*.*^49^ showed that probiotic did not have a significant effect on the digestibility of DM. Moreover, the increased Ca absorption and retention associated with probiotic supplementation^31^ could explain the improvement in egg weight and eggshell quality observed in the current study. Mikulski et al*.*^10^ stated that probiotic inclusion had no effect on metabolizable energy and P retention. But, in hens fed diets supplemented with *P. acidilactici*, a significant increase in Ca retention was seen by an improvement in eggshell quality in the PA group. While egg weight is a largely heritable quality, the beneficial effect of probiotic feeding on egg weight and eggshell quality could be attributed to a more favorable intestinal environment that aids in nutrient digestibility^50,51^. Calcium and phosphorus are essential nutrients involved in many biological processes. These minerals are the most considerable in the body, with 99% of Ca and 80% of P stored as hydroxyapatite in the skeleton, and they both play a role in bone growth and mineralization^51^. The remaining Ca is found in extracellular fluid, plasma, and cells, where it is important for metabolism, blood coagulation, enzyme activation, neuromuscular function, muscle contraction, cell adhesion, and intracellular signaling. Two epithelial Ca selective anion channels^35,36^ that belong to the superfamily of transient receptor potential channels (TRPV5 and TRPV6)^37^ enable the initial step of active intestinal Ca transport-entry across the cell wall.

**Figure 5:**
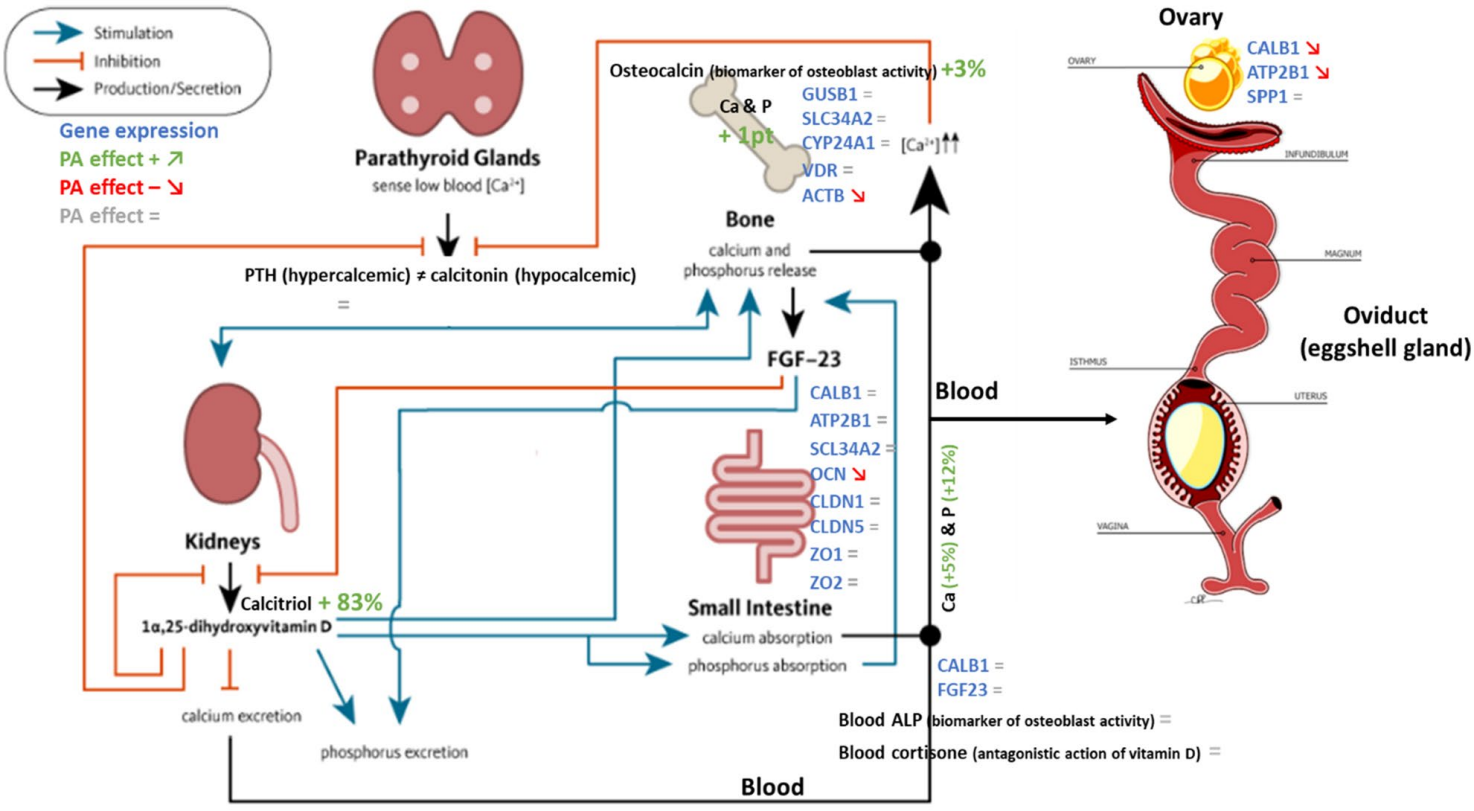
The different biomarkers and gene expression analyzed in the current study and the differential effects of PA supplementation on their level or expression (+, −, =) (adapted from https://lpi.oregonstate.edu/book/export/html/159).

Regarding the blood biomarkers related to Ca and P metabolism (osteocalcin, calcitriol, parathyroid hormone: PTH, alkaline phosphatase: ALP, Ca, P) and gene expression in different tissues (small intestine, ovarian tissue, bone and blood) at the start and end of the trial, several of them have been significantly impacted by the dietary treatment. Blood calcitriol, which is the active form of vitamin D (also known as 1,25-dihydroxycholecalciferol, 1.25(OH)2D3, promoter of Ca absorption) and blood osteocalcin (biomarker of osteoblastic activity) were increased respectively by 83% and 3% with PA in the current study, whereas there was no difference between the control and PA groups for ALP and PTH. Blood calcitriol concentration was increased following PA supplementation in the current study, providing an explanation for increased blood Ca (+ 5%) and P (+ 12%) levels PA in supplemented birds, both coming from stimulated intestinal absorption on one hand and increased bone release on the other hand. In birds, the efficient mechanisms for Ca^2+^ transfer from feed to the eggshell are under the control of 3 major Ca-regulating hormones: the hormonally active vitamin D metabolite (calcitriol), calcitonin and PTH^52^. Blood Ca^2+^ and blood calcitriol concentrations having in turn negative feedbacks on PTH hormone (Fig. 5), it could explain that its level was not modified following PA supplementation, allowing then to repress and/or modulate its hypercalcemic and bone resorption-inducing effects.

Osteocalcin has been used as a biomarker of osteoblast activity and bone formation/mineralization for evaluating bone remodeling in humans and rodents. Blood osteocalcin was increased by 3% in the current study following PA supplementation, raising the hypothesis of increased bone turnover, which could be in line with elevated observed levels of blood Ca and P in PA group. It would have been interesting to measure osteocalcin in bone in parallel to blood, to have a better insight into bone synthesis/resorption balance following PA supplementation, as elevated blood Ca (+ 5%) and P (+ 12%) levels were observed along with parallel increase of bone Ca and P (+ 1 pt) in PA group, suggesting fine-tuning process of bone metabolism (synthesis/resorption) in the case of the probiotic supplementation. However, as mean serum osteocalcin concentration was demonstrated to decrease with birds’ age, increased blood osteocalcin concentration observed in these aged hens following PA supplementation could be seen as a positive indicator of bone metabolism and health.

Besides blood biomarkers, PA significantly decreased or tended to decrease the expression of the following genes at the end of the trial: occludin in the small intestine, calbindin 1 (CALB1) in the ovary and actin B in the bone. As calcitriol was significantly increased (+ 83%) following PA supplementation, it can be hypothesized that negative feedback loops could have occurred to maintain Ca homeostasis by decreasing CALB1 expression in the ovarian tissue. It could also be hypothesized that decreased tight junction proteins expression (occludin) could induce better paracellular Ca transport from the gut lumen. Alternatively, PA could have induced lower gut inflammation, lowering then the need of gut epithelial barrier protection and/or renewal. The decrease of actin B-coding gene in the bone following PA supplementation could support a stimulated bone mineralization as illustrated by an enhanced bone resistance. Gene expression in the uterus (eggshell gland) was missing in this trial and it would have brought additional information regarding the effective Ca and P transport to the eggshell during its formation and mineralization. It would have also been interesting to analyze biomarker(s) of bone resorption or bone osteoclast activity, besides bone accretion and synthesis and bone osteoblast activity markers (ALP, osteocalcin). In the present study, relative mRNA expression of *GUSB1*, *SLC34A2*, *CYP24A1*, *VDR* and ACTB was similar among treatment groups. Our study's findings align with previous research of Mikulski et al.^10^, reinforcing the idea that the gut microbiota plays a critical role in bone health and calcium metabolism. These results underscore the potential for targeted interventions aimed at modulating the gut microbiome to prevent or mitigate bone-related diseases. Further research in this area may lead to novel probiotic therapies and dietary strategies for improving bone density and overall skeletal health.

## Conclusion

This study indicated that *P. acidilactici* CNCM I-4622 probiotic supplementation in laying hens’ diets has potential to improve zootechnical performance of the egg production, egg quality, bone strength (cohesiveness), bone calcium and phosphorus, intestinal morphology, nutrient digestibility of calcium and phosphorus, leading to increased blood Ca and P and also increased calcitriol level of relatively old layers, as well as egg freshness. In addition, CALB1 related gene in the ovarian tissue, occludin small intestine-related gene, and β- actin gene in the bone support the hypothesis that the paracellular pathway may be involved in Ca absorption in laying hens. It appears interesting to increase in parallel Ca and P in the blood and in the bone following PA supplementation, which then improved the mineral supply for eggshell formation without impacting bone integrity, and even increasing bone resistance. Several hypotheses could be raised to support the beneficial effects of this probiotic: (1) increased release of Ca and P (phytic acid) from plants, with increased Ca & P bioavailability, (2) higher Ca & P absorption by intestinal cells through local micro-acidification of the intestinal mucosa (HCl dissolves Ca), (3) oxido-reduction reactions related to the antioxidant/anti-inflammatory potential of the probiotic, (4) molecular effect on the activation of genes involved in Ca and/or in vitamin D metabolism, in hormonal response and in gut transport/integrity.

## Data Availability

All data generated in this study are included in the published article. The datasets generated during the current study are available from the corresponding author on demand upon reasonable request.
